# Effectiveness of BNT162b2 Vaccine in Adolescents during Outbreak of SARS-CoV-2 Delta Variant Infection, Israel, 2021

**DOI:** 10.3201/eid2711.211886

**Published:** 2021-11

**Authors:** Aharona Glatman-Freedman, Yael Hershkovitz, Zalman Kaufman, Rita Dichtiar, Lital Keinan-Boker, Michal Bromberg

**Affiliations:** The Israel Center for Disease Control, Ramat Gan, Israel (A. Glatman-Freedman, Y. Hershkovitz, Z. Kaufman, R. Dichtiar, L. Keinan-Boker, M. Bromberg);; Tel Aviv University, Tel Aviv, Israel (A. Glatman-Freedman, M. Bromberg); Haifa University, Haifa, Israel (L. Keinan-Boker)

**Keywords:** 2019 novel coronavirus disease, coronavirus disease, COVID-19, severe acute respiratory syndrome coronavirus 2, SARS-CoV-2, viruses, respiratory infections, zoonoses, vaccine effectiveness, Delta variant, adolescents, Israel

## Abstract

In Israel, the BNT162b2 vaccine against severe acute respiratory syndrome coronavirus 2 was approved for use in adolescents in June 2021, shortly before an outbreak of B.1.617.2 (Delta) variant–dominant infection. We evaluated short-term vaccine effectiveness and found the vaccine to be highly effective among this population in this setting.

In May 2021, the US Food and Drug Administration and the European Medicines Agency expanded existing authorization for BNT162b2 vaccine (Pfizer-BioNTech, https://www.pfizer.com) against severe acute respiratory syndrome coronavirus 2 (SARS-CoV-2) to include its use in adolescents 12–15 years of age (*1*,*2*). On June 2, 2021, the Israel Ministry of Health declared the availability of BNT162b2 vaccine for adolescents 12–15 years of age (*3*) as a 2-dose regimen, given 21 days apart. By August 26, 2021, a total of 277,218 adolescents (46.1% of those eligible) had received 1 dose of the vaccine and 187,707 (31.2%) had received 2 doses (Figure 1, panel A). In mid-June 2021, after a month of extremely low SARS-CoV-2 activity in Israel, 2 local outbreaks erupted (*4*–*6*). These outbreaks marked the beginning of a new widespread SARS-CoV-2 outbreak in Israel (Figure 1, panel B), dominated by the B.1.617.2 (Delta) variant, which accounted for 93%–99% of the sequenced viruses during July and August 2021 (*7*). We analyzed effectiveness of this vaccine among adolescents who had been vaccinated in the early stages of this outbreak in Israel. The study was approved by the superior ethical committee of the Israel Ministry of Health and included exemption from informed consent.

**Figure 1:**
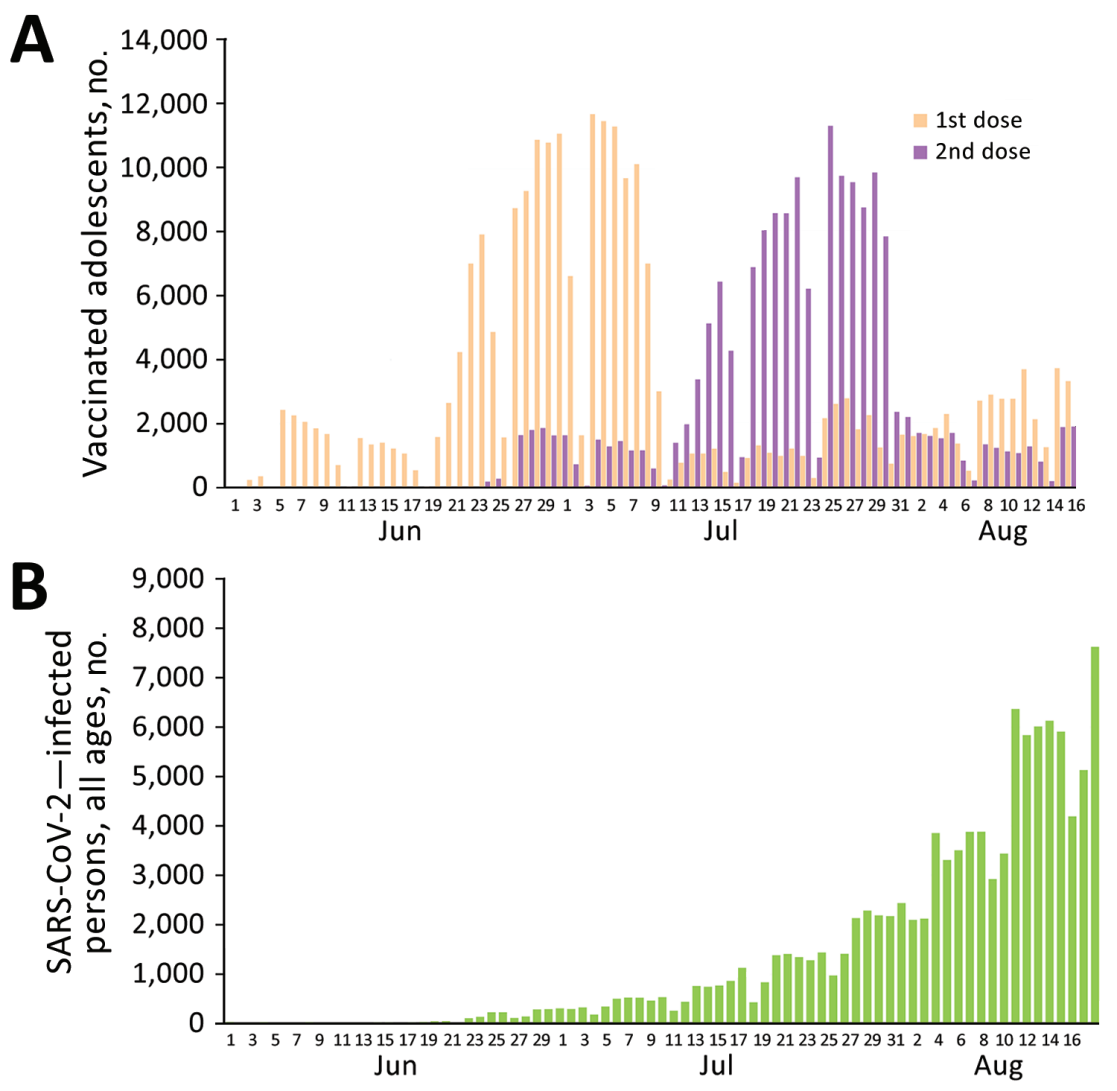
Vaccine doses among adolescents and total severe acute respiratory syndrome coronavirus 2 infections, Israel, June 1–August 26, 2021. A) Daily frequency of administration of first and second dose of BNT162b2 vaccine (Pfizer-BioNTech, https://www.pfizer.com) among adolescents 12–15 years of age. B) Daily cases of severe acute respiratory syndrome coronavirus 2 infection in persons of all ages.

## The Study

We performed a nationwide retrospective cohort study to estimate vaccine effectiveness against PCR-confirmed SARS-CoV-2 infections among adolescent Israel residents 12–15 years of age who had received the second vaccine dose during July 1–24, 2021. The data sources used are described in detail elsewhere (*8*). We estimated vaccine effectiveness and 95% CIs by using (1 – incidence rate ratio) × 100 for 1–7, 8–14, 15–21, and 22–28 days after the second vaccine dose. Incidence rate ratio denotes the ratio of the rate of PCR-confirmed SARS-CoV-2 infections in the vaccinated and unvaccinated groups.

We excluded from analysis adolescents who had had a documented SARS-CoV-2–positive PCR result before the evaluation periods, regardless of their vaccination status. When several positive SARS-CoV2 test results were documented for the same person during the study period, we included only the first result in our analysis.

We determined the number of unvaccinated controls for each date during July 1–24, 2021, by omitting the number of fully vaccinated adolescent Israel residents 12–15 years of age who had received the second BNT162b2 vaccine dose on a particular date from the total number of Israel residents who did not have a documented SARS-CoV-2–positive test result and had not received only a single vaccine dose by that date. We expressed the denominators of the vaccinated and unvaccinated groups in person-days.

**Figure 2:**
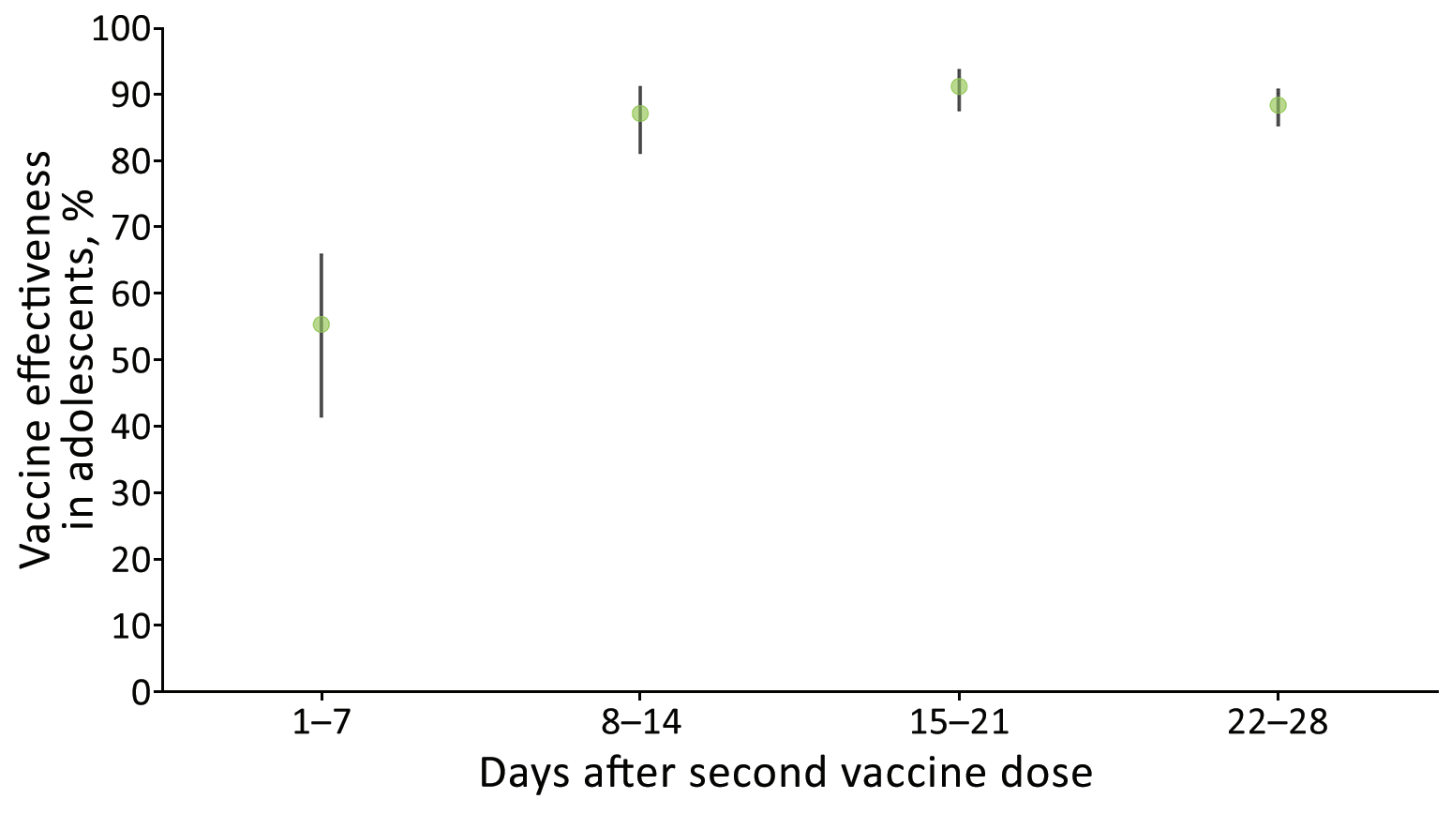
Vaccine effectiveness against severe acute respiratory syndrome coronavirus 2 infection in adolescents 12–15 years of age, by time after second dose of BNT162b2 vaccine (Pfizer-BioNTech, https://www.pfizer.com), Israel, 2021. Error bars indicate 95% CIs.

After administration of the second vaccine dose, crude vaccine effectiveness against laboratory-confirmed SARS-CoV-2 infection was 55.3% (95% CI 41.3%–66.0%) in the first week, 87.1% (95% CI 81.0%–91.2%) in the second week, 91.2% (87.4%–93.8%) in the third week, and 88.2% (95% CI 85.0%–90.7%) in the fourth week (Table; Figure 2). Vaccine effectiveness differed significantly between the first and subsequent weeks, but we found no statistically significant differences in vaccine effectiveness among the second, third, and fourth weeks. Because of the small number of cases of SARS-CoV-2 infection among vaccinated adolescents, we could not adjust for weekly vaccine effectiveness evaluation. However, adjustments for sex and epidemiologic week for days 8–28 after the second dose combined demonstrated adjusted vaccine effectiveness of 91.5% (95% CI 88.2%–93.9%) against SARS-CoV-2 infection (Table). We did not estimate vaccine effectiveness against symptomatic diseases because epidemiologic investigation was performed for 42% of vaccinated and 40% of unvaccinated adolescents in our cohort.

**Table:** Effectiveness of BNT162B2 vaccine against PCR-confirmed SARS-CoV-2 infection in adolescents 12–15 years of age after receipt of second dose, Israel, 2021*

Period	Days after second dose	Unvaccinated, SARS-CoV-2–positive, no.	Unvaccinated person-days	Vaccinated, SARS-CoV-2–positive, no.	Vaccinated person-days	Crude vaccine effectiveness (95% CI)	Adjusted vaccine effectiveness (95% CI)
Week 1	1–7	1,825	10,148,829	53	673,129	55.3 (41.3–66.0)	NA
Week 2	8–14	2,923	9,750,816	26	672,790	87.1 (81.0–91.2)	NA
Week 3	15–21	4,906	9,386,429	31	672,624	91.2 (87.4–93.8)	NA
Week 4	22–28	7,510	8,905,457	67	672,328	88.2 (85.0–90.7)	NA
Weeks 2–4	8–28	8,144	13,623,714	124	2,034,591	89.8 (87.8–91.5)	91.5 (88.2–93.9)

*BNT162B2 vaccine, Pfizer-BioNTech (https://www.pfizer.com) NA, not applicable; SARS-CoV-2, severe acute respiratory syndrome coronavirus 2.

As of August 26, 2021, none of the vaccinated adolescents who became SARS-CoV-2–positive on days 1–28 after the the second vaccine dose had been hospitalized. By that same date, among unvaccinated adolescents, 7 (0.38%) of 1,825 who tested positive for SARS-CoV-2 on days 1–7 after vaccinated adolescents had received their second vaccine dose and 26 (0.32%) of 8,144 who tested positive on days 8–28 were hospitalized. Also by August 26, no vaccinated or unvaccinated SARS-CoV-2–positive adolescents had died. 

## Conclusions

The BNT162b2 vaccination campaign for adolescents 12–15 years of age in Israel coincided with the outbreak of the SARS-CoV-2 Delta variant. As such, the timing enabled estimation of vaccine effectiveness against SARS-CoV-2 infection for this age group during predominant circulation of the Delta variant.

Our results demonstrate high vaccine effectiveness against SARS-CoV-2 infection in this population starting the second week after the second vaccine dose. These estimates are somewhat lower than those that had been estimated for persons 16–39 years of age during the same time intervals after the second vaccine dose during circulation of the SARS-CoV-2 Alpha variant and wild-type virus in Israel (*8*). Specifically, adjusted vaccine effectiveness against SARS-CoV-2 infection for persons 16–39 years of age was 93.2% (95% CI 91.9%–94.2%) at 8–14 days, 96.7% (95% CI 95.8%–97.4%) at 15–21 days, and 96.6 (95% CI 95.7%–97.3%) at 22–28 days after receipt of the second vaccine dose. Although vaccine effectiveness estimates and 95% CIs during the circulation of the Alpha variant and the wild-type virus were adjusted for age, sex, and epidemiologic week, only minor differences in point estimates and 95% CIs were noted between crude and adjusted vaccine effectiveness (*8*).

The effectiveness estimate of 55.3% in the first week after the second dose probably reflects the effect of the first vaccine dose. This estimate is consistant with previous estimates of vaccine effectiveness 14–20 days after the first dose (*8*–*10*).

Our findings are consistent with those of a recent study from the United Kingdom, which demonstarated vaccine effectiveness of 88.0% (95% CI 85.3%–90.1%) against symptomatic disease caused by the SARS-CoV-2 Delta variant (*11*), compared with vaccine effectiveness of 93.7% (95% CI 91.6%–95.3%) against disease caused by the Alpha variant among persons >16 years of age who had received 2 doses of BNT162b2 (*11*). However, that study addressed neither the interval between the 2 doses nor the exact interval between assessment of vaccine effectiveness and the date of the second dose (*11*).

As of September 2021, two controlled studies had assessed vaccine efficacy in adolescents, without specifying the SARS-CoV-2 variant (*12*,*13*). One study reported BNT162b2 vaccine efficacy of 100% against laboratory-confirmed COVID-19 >7 days after receipt of the second vaccine dose at 21 days after the first dose (*12*). The other study reported that vaccine efficacy of the mRNA-1273 vaccine 14 days after the second dose was difficult to assess because of the low incidence of laboratory-confirmed COVID-19 in the trial population (4 cases in the placebo group and 0 cases in the mRNA-1273 group) (*13*). Of note, the geometric mean ratio of neutralizing antibodies in adolescents receiving those vaccines was similar to or greater than that of young adults after receipt of 2 doses (*12*,*13*).

Behavioral and testing policy factors can potentially affect estimations of vaccine effectiveness. Behaviors that increase exposure to SARS-CoV-2 may be assumed by vaccinated or unvaccinated adolescents for different reasons and are difficult to measure. During the study period, SARS-CoV-2 testing was available in Israel regardless of vaccination status.

The recent rise in SARS-CoV-2 cases in Israel raised 2 concerns. The first concern was that BNT162b2 vaccine–elicited immunity was waning. Waning of spike protein antibody levels was detected over time after receipt of a second dose of SARS-CoV-2 vaccines (*14*). The second concern was that the vaccine was not effective against the SARS-CoV-2 Delta variant. However, our findings indicate that the BNT162b2 vaccine provides adolescents with highly effective short-term protection against the SARS-CoV-2 Delta variant. 
